# Transesophageal electrophysiology study in the diagnosis of dual atrioventricular nodal nonreentrant tachycardia

**DOI:** 10.1111/anec.12845

**Published:** 2021-03-30

**Authors:** Jing Chen, Fan Lin, Ping Zuo, Shixu Li, Li Lin, Bei Wang, XiaoYun Yang

**Affiliations:** ^1^ Division of Cardiology and Department of Internal Medicine TongJi Hospital TongJi Medical College Huazhong University of Science and Technology Wuhan China; ^2^ Department of Cardiology, People's Hospital of Badong County Badong County China; ^3^ Department of Rheumatology and Immunology TongJi Hospital TongJi Medical College Huazhong University of Science and Technology Wuhan China

**Keywords:** 1:2 AV conduction, ablation, ECG, transesophageal electrophysiological study

## Abstract

“Double fire” is generally characterized by 1:2 atrioventricular conduction of sinus beats traveling down fast and slow pathways that result in double ventricular response. When this phenomenon repeats rapidly, dual atrioventricular nodal nonreentrant tachycardia (DAVNNT) occurs. We report a case of an irregular tachycardia with a comprehensive record that includes an electrocardiogram, a transesophageal electrophysiology study, and an intracardiac electrophysiology study. This is the first report of transesophageal electrophysiology study in the diagnosis of DAVNNT. A diagnosis of DAVNNT was deduced, and the patient was successfully treated with radiofrequency ablation of the slow pathway.

## 1 INTRODUCTION

The phenomenon of dual fast and slow AV conduction of single atrial beat was first described in 1975 (Wu et al., 1975), and a number of case reports and small case series have been published over the past few years (Peiker et al., 2016; Wang, 2011). Dual AV node physiology provides the substrate for a sinus beat resulting in a double ventricular response (Wang, 2011). DAVNNT is associated with a reversible tachycardia‐induced cardiomyopathy (Clementy et al., 2007; Gonzalez‐Torrecilla & Avila‐Alonso, 2019). Slow pathway ablation is the main treatment for this type of tachyarrhythmia. Right atrial pacing has been used to successfully suppress DAVNNT when slow pathway ablation failed (Wang et al., 2013). Patient characteristics and clinical presentation are not helpful in discriminating between DAVNNT and more common arrhythmias. Supraventricular tachycardias such as atrial fibrillation or atrial tachycardia are common erroneous differential diagnosis. DAVNNT was also misdiagnosed as junctional extrasystoles, ventricular premature beats, and atypical AVNRT.

Transesophageal cardiac electrophysiological study (TE‐EPS) is a noninvasive approach for clinical electrophysiological diagnosis and treatment. TE‐EPS can be applied in (a) evaluation of the function of sinoatrial node including the sinoatrial node recovery time and sinoatrial conduction time to confirm the presence of sick sinus syndrome; (b) measurement of the refractory periods of the atria and ventricles; (c) diagnosis, induction, and termination of supraventricular tachycardia; (d) pace the heart temporally in patients with severe bradycardia in the ward in urgent situations; and (e) perform heart pacing loading test in patients who are not suitable for exercise test. We present the first report of TE‐EPS for the assistance in the diagnosis of a DAVNNT.

## 2 CASE REPORT

**Figure 1:**
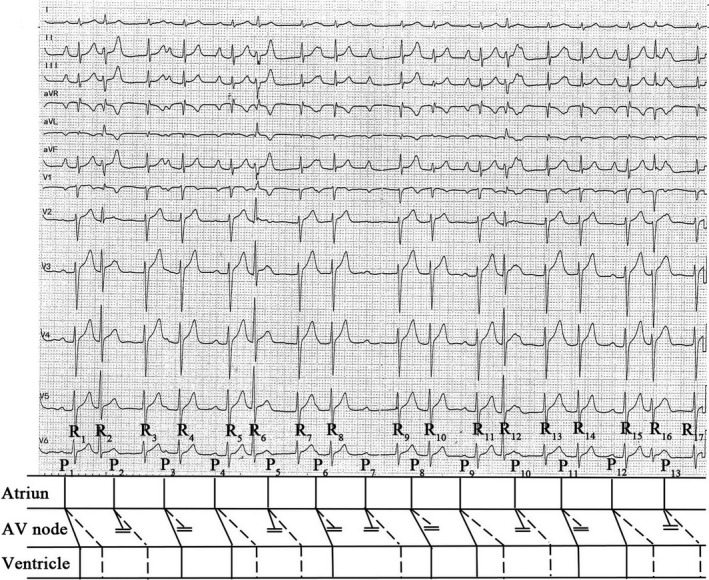
Patient's first ECG and corresponding schematic ladder diagrams with AV conduction. Two antegrade conduction AV pathways are indicated by *straight lines* (fast pathway) and *dotted lines* (slow pathway)

**Figure 2:**
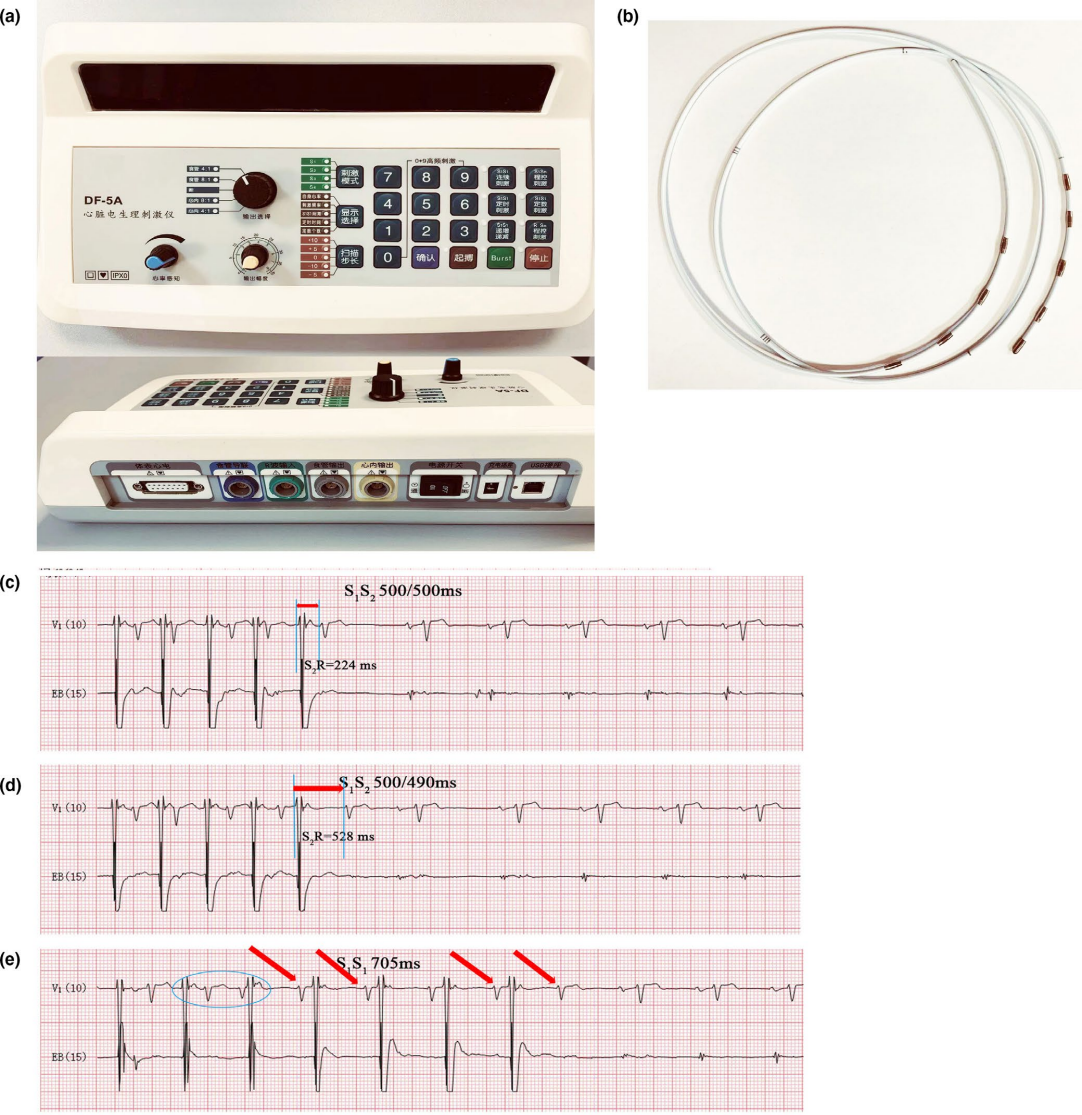
Atrial programmed stimulation in a transesophageal electrophysiological study. (a) Cardiac electric stimulator. (b) Esophageal diagnostic catheter. (c) When S1S2 was 500/500 ms, the S2‐QRS interval was 224 ms. (d) When S1S2 was 500/490 ms, the S2‐QRS interval was 528 ms. (e) When atrial pacing with an S1S1 interval of 705 ms, the P wave linked to two QRS complexes was observed. EB (15) represents that the amplitude of the EB lead is 15 mm/mV

**Figure 3:**
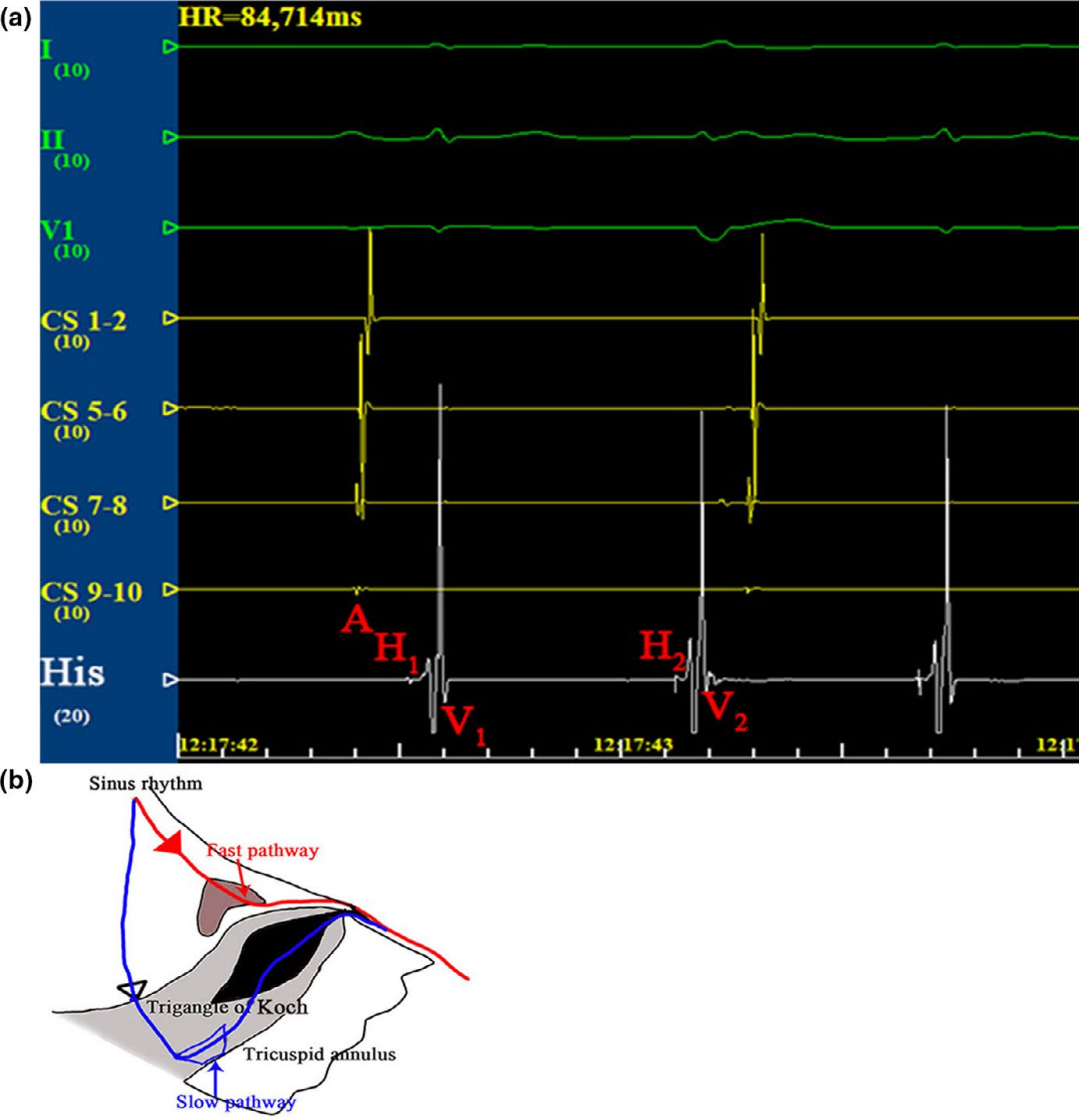
Intracardiac tracings from an electrophysiology study. (a) Intracardiac tracing reveals 1:2 AV conduction with a pattern A‐H_1_‐V_1_‐H_2_‐V_2_ (paper speed at 100 mm/s). (b) Schematic diagram of the mechanism of DAVNNT. AH, interval between the right atrium and His bundle. HV, interval between the His bundle area and ventricle

A 56‐year‐old male patient had been experiencing intermittent palpitation and discomfort for over 6 years which was worsening over the past several months. He visited to an emergency department for first‐time consultancy 3 months ago and was diagnosed with atrial fibrillation based on the ECG in Figure 1. The echocardiographic examination revealed arrhythmia and ruled out structural heart disease. The patient did not take medication and then referred to us for further treatment and potential ablation for atrial fibrillation. The first 12‐lead electrocardiogram (ECG) is shown in Figure 1. The patient was subjected to TE‐EPS (Figure 2) and electrophysiologic study with His bundle recordings (Figure 3).

The baseline ECG shows intermittent P waves (P_1_, P_4_, P_9_, P_12_) linked to the first of the two QRS complexes with a constant PR interval of 220 ms (Figure 1). By a beat‐to‐beat morphological analysis of T waves, hidden P waves (P_2_, P_5_, P_13_) were then identified (Figure 1). Subsequently, regular PP (P_1_–P_13_) intervals were identified with a cycle length of 760 ms. Regular P waves are upright in the leads I, II, aVF, and V4‐V6 and inverted in lead aVR, suggesting they are sinus P waves. The presence of regular sinus rhythm without other apparent atrial activity excludes a diagnose of AF. Therefore, the possible differential diagnosis from the data shown in Figure 1 includes (a) sinus rhythm with ventricular extrasystoles, (b) sinus with junctional extrasystoles, (c) sinus rhythm with dual atrioventricular (AV) nodal pathways, and (d) atrial tachycardia.

Furthermore, PR interval prolongation (440 ms) after the 2nd QRS complexes (R_2_, R_6_, R_12_, R_16_) (Figure 1) can be interpreted in two ways: (a) concealed retrograde conduction to the AV node by ventricular or junctional extrasystoles which prolongs the ensuing AV node conduction and (b) by the AV node slow pathway conduction (Figure 1). The record shows both narrow and wide irregular QRS. The ladder diagram in Figure 1 reveals the AV conduction.

TE‐EPS provided an important diagnose basis of our patient. Oral administration of dyclonine hydrochloride mucilage was applied before TE‐EPS. The 10 ml dose was orally administered: The first 5 ml was swallowed slowly to allow observation of possible allergic reaction, and the second 5 ml was maintained in the mouth for 5–10 min before the TE‐EPS. The esophageal diagnostic catheter was used for recording the electrogram in esophagus and usually has four electrodes in pairs in both ends, and its diameter is usually 6 French or 7 French (Figure 2b). Its output can be adjusted from 0 to 50 V and pulse width 0–10 ms. Due to the close proximity, the P waves can be clearly recorded by the catheter electrode placed in the esophagus.

After the catheter insertion, programmed stimulations were started. Cardiac electric stimulator (DF‐5A, Suzhou Dongfang Electronic Instrument Factory, Suzhou, China) of TE‐EPS can generate programmed stimulation or burst pacing (Figure 2a). The pacing stimulus is comprised of a train of stimuli (S1) at a constant cycle length and a progressively premature extrastimulus (S2) succeeding a train of stimuli (S1) delivered in the atrium. The S1S2 is composed of four S1 and one S2 stimuli. S1S1 cycle length and the starting coupling interval were set to 500 ms. When the S1S2 interval was 500 ms, transesophageal atrial pacing captured the left atrium, and S2‐QRS interval was approximately 224 ms. When the S1S2 interval was reduced to 490 ms (Figure 2c), decremental conduction appeared in the AV node and resulted in a prolongation of S2‐QRS interval to approximately 528 ms. An increase in the S2‐QRS interval greater than 50 ms in response to a decrease in the S1S2 coupling interval of 10 ms is defined as a discontinuous AV nodal function curve and taken as evidence of dual anterograde AV pathways (Bagliani et al., 2018).

When the S1S1 cycle length was 705 ms, the P waves were linked to two QRS complexes (blue circle), and the excitation subsequently conducted to the ventricle via the slow conduction pathway (red arrows) (Figure 2d). Atropine and isoproterenol are expected to exacerbate triggered junctional activity and suppress DAVNNT and have been suggested as a means to differentiate the two (Csapo, 1979). Atropine produces a significant reduction in the effective refractory period of the slow and fast conducting pathways. Improvement in antegrade as well as retrograde AV nodal conduction encourages the occurrence of atrial echoes and the induction of stable junctional tachycardias (Neuss et al., 1975; Wu et al., 1979). Our patient had no sustained simultaneous anterograde fast and slow pathway conduction after the administration of atropine, probably because the drug had improved antegrade fast pathway conduction.

Finally, the patient was subjected to an electrophysiology study (EPS). During EPS, a decapolar coronary sinus (CS) catheter was placed into the coronary sinus. The proximal pair (CS9–10) of electrodes was positioned at the coronary sinus ostium, and the distal pair (CS1–2) of electrodes was located at the lateral aspect of the great cardiac vein (Bagliani et al., 2018). The His potential was recorded by a catheter positioned across the tricuspid valve distally and more proximally. RV recordings were recorded by catheter positioned in right ventricle (Nogami, 2015).

Spontaneously tachycardia was recorded by Figure 3a. Atrial potentials recorded by catheters in the coronary sinus were regular with a cycle length of 847 ms. His potentials recorded by catheters showed double ventricular response with a pattern A‐H_1_‐V_1_‐H_2_‐V_2_. The AH_1_ and AH_2_ intervals were 118 and 718 ms, respectively, and H_1_V and H_2_V intervals were constant at 37 ms (Figure 3a). Junctional extrasystoles may double the ventricular rate; however, they typically have a less predictable coupling interval to the receding QRS complex or His potential (Peiker et al., 2016; Wang, 2011). Because of the fixed interval between His and ventricular activation, junctional extrasystoles are unlikely.

In RAO (right anterior oblique), we advance the ablation catheter into the ventricle to have more V than A, near and initially downward into the coronary ostium region. To prevent fast pathway damage and an AV block, we looked carefully for His signals in the ablation catheter before starting ablation. The region of ablation was at the inferior (posterior) part of the tricuspid annulus where A/V ratio <1. We start slow pathway ablation at 35W using a nonirrigated 4‐mm catheter and 15–20 s for each application waiting for a junctional rhythm to appear. When the junctional rhythm appeared, we continued ablation for another 30–60 s. After radiofrequency (RF) catheter ablation was performed in the slow pathway, the dual ventricular response disappeared. CS pacing showed no evidence of the dual AV node pathway or the onset of tachycardia in a 30‐min observation. RV pacing also shows no VA retrograde conduction.

The patient was reexamined with a Holter ECG after radiofrequency catheter ablation. Holter monitoring reveals average heart rate (HR) that was significantly decreased to 68 beats/min (before the ablation, it was 92 beats/min), and maximum and minimum HR of 103 and 54 beats/min, respectively.

## 3 DISCUSSION

1:2 atrioventricular conduction occurs when the atrial impulse uses both pathways at the same time; the fast pathway impulse depolarizes ventricles, and then, the slow pathway impulse arrives after the ventricular refractory period (Figure 3b). Our case first reported the utility of TE‐EPS in diagnose of DAVNNT.

Our hospital is a large size Tertiary comprehensive hospital and has one of the largest cardiac function examination centers in China. Our cardiac function examination center is responsible for routine ECG, Holter ECG, TE‐EPS, follow‐up of patients with cardiac pacemaker, treadmill exercise test, and other examinations about noninvasive cardiac functional evaluation. Our center contains about 30 doctors majored in cardiology and more than 30 nurses. TE‐EPS in our center is completed by two cardiologists and two nurses in an independent examination room. The examination room is equipped with defibrillators. Two cardiologists are responsible for esophageal diagnostic catheter insertion and programmed stimulations. Two nurses are responsible for oral anesthetic and intravenous administration.

Our cardiac function examination center has performed TE‐EPS on 1,170 patients in 2020. For those patients who have financial concerns or an invasive test phobic or require dynamic observation and follow‐up, TE‐EPS is an alternative strategy. The cost of the EPS excluding the ablation is the 15 times cost of TE‐EPS.

TE‐EPS is contraindicated in the following situations: acute upper respiratory tract inflammation, large aortic aneurysm, severe hypertension or uncontrolled hypertension, severe esophageal disease and severe cardiac disease, cachexia, or other status, which the patient is intolerant to catheter insertion (Proietti et al., 2019).

As a diagnostic tool, TE‐EPS has some limitations. One of the most important limitations of TE‐EPS is the lack of tolerable ventricular stimulation in the majority of patients and the lack of His bundle recording, which has a crucial role in the diagnosis of some arrhythmias. Although the absence of His bundle recording, TE‐EPS can identify the dual AV pathway, minimize the possibility of junctional extrasystoles, and provided a screening before a consequent invasive electrophysiology study.

TE‐EPS is a safe and noninvasive method and provides indications for ablation. TE‐EPS also provides a relatively noninvasive means of risk stratification. It is also valuable for patients with typical symptom of tachycardia, but there is no any ECG at the time of attack, or the type of tachycardia is not clearly identified by ECG. As for our patient, the routine ECG revealed the several possibilities of tachycardia. The noninvasive TE‐EPS examination provides a preliminary tachycardia screening.

## 4 CONCLUSION

TE‐EPS provides an important electrophysiology basis before invasive EPS. It can be applied for screening and provide the ambulatory observation during consultations.

## CONFLICT OF INTERESTS

All authors declare no conflicts of interest. We confirm that the manuscript has been read and approved by all named authors and that there are no other persons who satisfied the criteria for authorship but are not listed. We further confirm that the order of authors listed in the manuscript has been approved by all of us.

## Data Availability

All data generated or analyzed during this study are included in this article.
